# Artificial-intelligence-assisted mass fabrication of nanocantilevers from randomly positioned single carbon nanotubes

**DOI:** 10.1038/s41378-023-00507-1

**Published:** 2023-03-22

**Authors:** Yukihiro Tadokoro, Keita Funayama, Keisuke Kawano, Atsushi Miura, Jun Hirotani, Yutaka Ohno, Hiroya Tanaka

**Affiliations:** 1 Toyota Central R&D Labs., Inc., Nagakute, Aichi Japan; 2 Toyota Research Institute of North America, Ann Arbor, MI USA; 3 Nagoya University, Nagoya, Aichi Japan

**Keywords:** NEMS, Electrical and electronic engineering, Carbon nanotubes and fullerenes

## Abstract

Nanoscale cantilevers (nanocantilevers) made from carbon nanotubes (CNTs) provide tremendous benefits in sensing and electromagnetic applications. This nanoscale structure is generally fabricated using chemical vapor deposition and/or dielectrophoresis, which contain manual, time-consuming processes such as the placing of additional electrodes and careful observation of single-grown CNTs. Here, we demonstrate a simple and Artificial Intelligence (AI)-assisted method for the efficient fabrication of a massive CNT-based nanocantilever. We used randomly positioned single CNTs on the substrate. The trained deep neural network recognizes the CNTs, measures their positions, and determines the edge of the CNT on which an electrode should be clamped to form a nanocantilever. Our experiments demonstrate that the recognition and measurement processes are automatically completed in 2 s, whereas comparable manual processing requires 12 h. Notwithstanding the small measurement error by the trained network (within 200 nm for 90% of the recognized CNTs), more than 34 nanocantilevers were successfully fabricated in one process. Such high accuracy contributes to the development of a massive field emitter using the CNT-based nanocantilever, in which the output current is obtained with a low applied voltage. We further showed the benefit of fabricating massive CNT-nanocantilever-based field emitters for neuromorphic computing. The activation function, which is a key function in a neural network, was physically realized using an individual CNT-based field emitter. The introduced neural network with the CNT-based field emitters recognized handwritten images successfully. We believe that our method can accelerate the research and development of CNT-based nanocantilevers for realizing promising future applications.

## Introduction

Nanoscale cantilevers (nanocantilevers) have been widely studied in recent decades. Their mechanical behavior provides enormous benefits in scientific and practical scenarios; e.g., tracing a shape/surface with a nanocantilever allows us to measure the structure of materials at the nanoscale, or even smaller scales^1–4^. A resonantly driven nanomechanical vibration^5–8^ can perform ultra-sensitive detection of various physical quantities (for example, force^9,10^, mass^11,12^, and electric/magnetic spin^13^), which has led to the development of a wide range of applications, including chemical/biological/inertial sensors^14–17^, nanomechanical computing^18–21^ and quantum information science^22–24^. The electric property of the nanocantilever has enabled the development of a nanoscale field emitter^25–30^, which is often employed as an electron source^31^ for flexible displays, X-ray computed tomography, and optical applications^32–34^. Particularly, when the nanocantilever (and nanobeam) is fabricated using a single carbon nanotube (CNT), its remarkable mechanical property enhances the sensitivity to a very high level^35–37^. Outstanding electrical and thermal conductivities also contribute to the development of excellent nanoscale emitters^38,39^, which can be combined with nanomechanical systems to create novel information systems such as nanoscale communication devices^40–43^.

Toward the development of field-emission devices incorporating CNT-based nanocantilevers, the efficient fabrication of this nanostructure should be prioritized. Several methods have been explored for this fabrication^39,44,45^. One major method is based on chemical vapor deposition (CVD)^33,39,46^, wherein straight single CNTs are grown at certain positions^47–49^. This type of CNT can be used as a nanocantilever (or nanobeam), which has been favorably utilized in several pioneering studies (for example, refs. ^11,35–37,50^). However, it generally shows low production efficiency, which is acceptable only in scientific and/or early-stage prototyping efforts that do not require more than several samples. Hence, other approaches have been explored to efficiently develop a massive nanocantilever^44,45^. An array of single CNTs was successfully fabricated using the dielectrophoresis method^51,52^. This emerging approach requires additional electrodes to place single CNTs at the desired positions, which requires further investigation. Although CVD-based methods can fabricate arrayed CNTs, this type of structure is unsuitable for developing a massive field emitter^31,53,54^. Such CNTs are typically bundled^55–57^ and slightly bent, conditions that are incompatible with use as nanocantilevers. A silicon-based massive nanocantilever^58^ and field emitter^59,60^ have been fabricated using a semiconductor process.

**Fig. 1:**
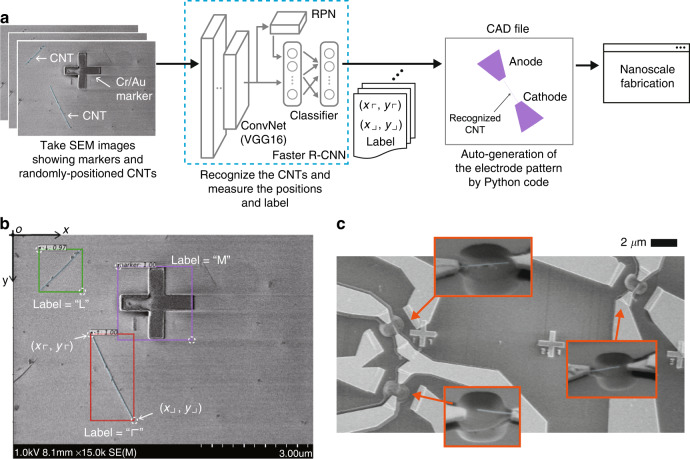
AI-assisted fabrication of nanocantilever and recognition/fabrication example. **a** Framework of the AI-assisted fabrication. The SEM image showing randomly positioned CNTs is input to the “Faster R-CNN^61^” neural network such that it can automatically recognize the CNTs. The recognized CNT is surrounded by a bounding box, after which the trained network outputs the positions of the top-left and bottom-right corners of the box, (*x*_*⌜*_, *y*_*⌜*_) and (*x*_*⌟*_, *y*_*⌟*_), respectively. In addition, this network decides which corner is preferable for positioning the anode, and the decision is applied to the label (see **b**). With this information, our Python code automatically generates an electrode pattern and creates a CAD file that is used in the nanoscale fabrication process. Finally, the massive nanocantilevers are simultaneously created, as shown in (**c**). **b** Example of the recognition result of randomly positioned CNTs. The trained network successfully recognizes both the CNT and the marker with the bounding box. The label “*⌜*” indicates that the top-left corner of the bounding box, which is one edge of the recognized CNT, is suitable for fabricating the anode. **c** Result of fabrication with the proposed AI-assisted method. Our method enables the creation of dense nanocantilevers in a limited space on a single substrate

Here, to efficiently fabricate massive field emitters with CNT-based nanocantilevers, we report a semiconductor-process-based method that utilizes artificial intelligence (AI). We used straight single CNTs, which fit the shape of the nanocantilevers. The CNTs were fabricated using the arc discharge method and dispersed in isopropyl alcohol (IPA); by dropping them onto a silicon substrate, we obtained many randomly positioned single CNTs. However, the problem with this approach is that the locations of the single CNTs on the substrate are unknown. To efficiently and accurately find and measure these positions, we developed an AI-assisted method; SEM images, which show the surface of the substrate, were input to a state-of-the-art deep neural network, Faster R-CNN^61^. The network trained on the original dataset automatically recognizes the CNTs, measures their position, and determines the edge of the CNT to be clamped with an electrode (see Fig. 1a). This information is used in the fabrication process employing an electron beam (EB) lithography to form the two electrodes (anode/cathode). Our proposed AI-assisted method significantly shortens the time required to find the CNTs and design the electrodes on them. Our experimental results showed that we successfully fabricated at least 34 nanocantilever-based field emitters in one fabrication process. These emitters contain a nanoscale gap between the emitter (the tip of the single CNT) and counter electrode, as designed. The length distribution of the nanocantilevers was within the expected range.

We also showed a beneficial application of our massive CNT-nanocantilever-based field emitters by applying them to neuromorphic computing. Recently, this type of computing framework (with neural networks) has attracted much attention due to its outstanding characteristics of high computational speed and low power consumption when compared with the traditional von Neumann architecture^62–71^. Prior work has shown that bulk CNTs with functional chemical molecules contribute to the realization of this novel computation framework^72^. We found that an individual CNT-based field emitter physically realized one of the key functions of the neural network, namely, the activation function^73^. The output current from the emitter exponentially responds to the applied voltage^29,39,42,60,74–76^. This typical nonlinear response realizes the activation function used in this study. Our proposed AI-assisted method enables us to investigate this computing application because it allows for the fabrication of more than several hundred emitters, as are required in the network. We emulate that our neural network with the CNT-nanocantilever-based emitters can recognize handwritten images successfully.

## Results and discussion

### AI-assisted fabrication of massive nanocantilevers

Our proposed AI-assisted fabrication process is presented in Fig. 1a. We attempted to fabricate massive nanocantilevers from randomly positioned CNTs in a limited area. The nanocantilever comprised a single CNT clamped by a cathode, with the counter-electrode of the anode receiving the electrons emitted from the tip of the CNT (the structure is depicted in Fig. S2b in the Supporting information). Tangled CNTs are first dispersed on the substrate (see the section “Materials and methods”). We then obtain SEM images that show each part of the resulting substrate. However, it is unknown which image contains the single CNTs and the specific locations of the CNTs. To automatically find and measure their positions, these images are input to the state-of-the-art neural network of “Faster R-CNN^61^.” The recognized CNT (and the marker) is surrounded by a bounding box, and the trained network outputs the positions of the top-left and bottom-right corners of the box, (*x*_*⌜*_, *y*_*⌜*_) and (*x*_*⌟*_, *y*_*⌟*_), respectively (see Fig. 1b). The network also determines which edge of the recognized CNT is suitable for the anode side. This information is found in the output label, obtained by training the network with our original dataset. These outputs are then used to automatically create CAD design files to fabricate the trapezoidal electrode pattern. Finally, a fabrication process (such as EB lithography) is used to form the two electrodes (anode/cathode) according to the information in this file. Note that the details of the fabrication process are provided in the “Materials and methods” section. This AI-assisted method significantly shortens the time required to find the CNTs and fabricate the electrodes over them.

Employing the two-stage object recognition method of Faster R-CNN provides outstanding capability in terms of recognition, position measurement, and labeling of the SEM images. The network consists of a convolutional network (CNN), region proposal network (RPN), and classifier, as shown in Fig. 1a. The CNN extracts the feature contained in the input image and outputs it to both the RPN and classifier. The RPN indicates the region in the picture where the target object of the CNT may be contained. This advantageous characteristic enhances the recognition ability of the classifier; two kinds of information are output by the network-the recognized position (within a bounding box) and the label. A neural network with these outputs is thus suitable for our goal. The recommended edge for fabricating the anode is obtained as the label, in addition to the position of the recognized CNT within the bounding box whose top-left and bottom-right corners are located at (*x*_*⌜*_, *y*_*⌜*_) and (*x*_*⌟*_, *y*_*⌟*_), respectively. The label ∈{“*⌜*”, “*⌝*”, “*⌞*”, “*⌟*”, “M”} contains two types of information—the recommended edge for the anode (“*⌜*”, “*⌝*”, “*⌞*”, “*⌟*”) and the marker (“M”); see the example shown in Fig. S1 in the Supporting information. For example, in Fig. 1b, the two edges of the recognized CNT, which is enclosed by the green box, are located at (*x*_*⌜*_, *y*_*⌟*_) and (*x*_*⌟*_, *y*_*⌜*_), and the label = “*⌞*” indicates that the anode should be fabricated around the edge (*x*_*⌜*_, *y*_*⌟*_). These positions are represented in pixels according to a coordinate system, with the origin *o* located at the top-left corner. The neural network structure is the same as described in the original study^61^, and the “Materials and Methods” section provides the details of the dataset used here and the training process of the network.

After successfully recognizing the single CNTs and obtaining the information shown in Fig. 1b, our Python code, which is based on the library gdsCAD^77^, automatically generates the pattern of the trapezoidal electrodes in a CAD file. According to the pre-designed shapes of the two electrodes, as shown in Fig. S2b in the Supporting information, a pattern is automatically designed with the central position (1), (2), and rotation angle *θ* of the electrodes (see “Materials and methods”). With these CAD files, the 110-nm-thick Au/Ti electrodes were fabricated through EB lithography and deposition. To form a hole around the nanocantilever, we also patterned the rectangular etching area centered at (*X*_Hole_, *Y*_Hole_) using EB lithography. This central position was calculated using (3) based on the information obtained with Faster R-CNN. The silicon dioxide under the single CNTs was then etched with buffered hydrofluoric acid. We finally developed the nanocantilever by supercritical drying of the substrate.

The result of the fabrication with the proposed AI-assisted method is shown in Fig. 1c. Our method enables the efficient fabrication of dense nanocantilevers from randomly positioned CNTs in a limited space on a single substrate. It is observed from Fig. 1c that the length and size of the gap differ among the fabricated nanocantilevers. Such structural variances strongly affect the performance of field emissions. In the next section, we present the fabrication performance, particularly the statistical performance in relation to CNT length and the gap between the CNT tip and its supporting structure. Note that in this figure, additional electrodes are connected to the nanocantilevers. These electrodes were used to measure the field-emission performances.

### AI-assisted fabrication performance

#### Recognition and structural performance

**Fig. 2:**
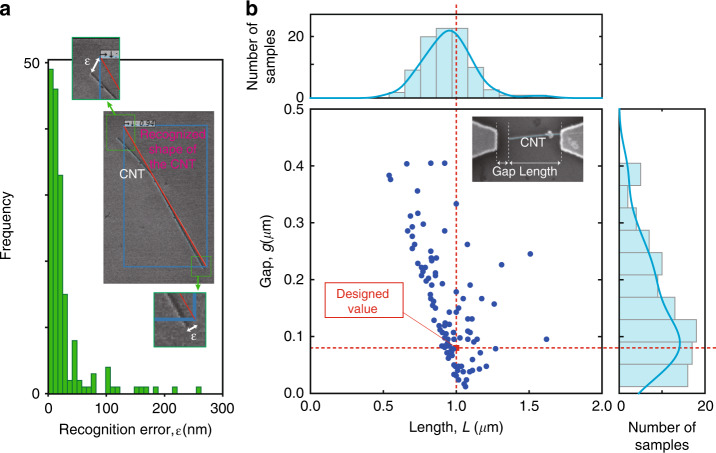
Recognition and fabrication performances of the CNT nanocantilevers. **a** Recognition performance of the randomly positioned CNTs. We manually inspected 96 fabricated samples using SEM to evaluate the recognition error *ε* (in nm), which represents the difference between the original and recognized edge positions of the CNT. We successfully measured the positions of ~60% of the recognized CNTs within the error *ε* ≤ 150 nm. **b** Measured gap and length, and corresponding histogram of the fabricated 101 samples. The designed values were *g* = 80 nm and *L* = 1.0 μm, which were the peak values of the measured gap and length; thus, we successfully fabricated many nanocantilevers. The discrepancy between the designed and measured value should originate from the recognition error, which is shown in (**a**)

We evaluated the recognition performance of the randomly positioned CNTs based on recognition error *ε* (in nm). This error describes the difference between the original and recognized edge position of a single CNT (see Fig. 2a). We manually inspected 96 recognized CNTs using SEM to measure the error in pixels, and the resulting histogram is depicted in Fig. 2a. To obtain this data, the error in nm was calculated by rescaling the measured error at 7.143 nm per pixel. We found that we successfully measured the position of approximately 60% of the recognized CNTs within the error of *ε* ≤ 150 nm. We designed the cathode with the upper base *W*_U_ = 300 nm (see Fig. S2b). This part holds the CNT to form the nanocantilever.

Even with the state-of-the-art AI-based recognition method of the Faster R-CNN, a large recognition error is observed in Fig. 2a. Indeed, although the median of *ε* is 125.5 nm, the average and standard variance of the recognition error were calculated as 567.4 and 1161.8 nm, respectively. These large values occur because the recognized position of the randomly positioned CNTs is far from the original position, which is caused by the deformation of the CNT and/or particles attached to the CNTs. These undesired structures may be eliminated by using CNTs with a clean surface and high crystallinity. Using a dataset containing a large deviation in the training process would make the resulting model more robust.

By using the AI-assisted fabrication method shown in Fig. 1a, we fabricated more than 8000 samples of nanocantilevers from the randomly positioned CNTs on a 1.0 mm square substrate. The designed structure is shown in Fig. S2 of the Supporting information; the designed values of the gap, length, upper base, and height of the electrode were *g* = 80 nm, *L* = 1.0 μm, *W*_U_ = 300 nm, and *H* = 1.0 μm, respectively. The proposed fabrication method drastically reduces the processing time necessary to recognize the randomly positioned single CNTs and design the electrodes using CAD. Indeed, the entire process required only 2 s, whereas manual recognition and designing required ~12 h.

After the fabrication, we manually inspected the 101 fabricated nanocantilevers using SEM to evaluate the resulting structural properties and the length and gap of the nanocantilevers. Figure 2b shows the scatter plots and histograms of these key properties (the curves over the histograms were drawn using kernel density estimation). We found that the histograms of the gap and length peaked at the designed values. This performance demonstrates that we successfully fabricated many nanocantilever-based nanoscale emitters. However, due to recognition error, there was a discrepancy between the designed and observed structures. A notable observation was that the erroneous points were bounded within the line, *g* + *L* = 1080 nm; in the automatic design on the CAD, the distance between the anode and cathode (*g* + *L*) was fixed to 1080 nm. Even with this recognition error, the points are not plotted in the left-side region of the line. Note that in Fig. 2b, the points are slightly shifted beyond this line because of the measurement error in the manual observation from the SEM images and because of deviations when preparing the electrodes with EB lithography. The large discrepancy from the designed value, particularly in the case of the large length *L* ≳ 1.1 μm, occurred when the leg part of the trapezoidal electrode held the CNT.

#### Field emission performance of the fabricated device

**Fig. 3:**
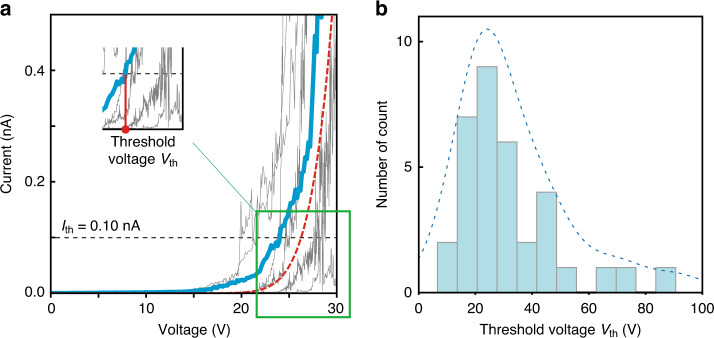
Field-emission performance of mass-fabricated CNT-nanocantilever-based emitters. **a** Measured I–V characteristics of nine samples of the fabricated emitters (gray and blue curves). We found deviations in the curves depending on the device, which were attributed to structural variations, such as at the gap shown in Fig. 2b. **b** Histogram of the threshold voltage *V*_th_ at which a sufficient amount of the induced field-emission current *I*_th_ = 0.10 nA is observed. The peak in this histogram should correspond to the maximum in Fig. 2b, where the gap and length of the fabricated emitters are of the designed values. According to this peak (*V*_th_ = 26.20 and *I*_th_ = 0.10 nA), we also plotted the theoretical curve shown in red in (**a**). This theoretical curve adequately captures the trend of the measured currents, showing that the measured currents were excited by the field emission

We evaluated the fabricated CNT-nanocantilever-based emitters in terms of field-emission performance. Due to the ultra-small gap (~80 nm) between the tip of the randomly positioned CNT and the anode, we observed a quantum phenomenon of field emission: a current was excited by applying a voltage between the anode and cathode. We successfully measured this current on 34 fabricated emitters by adopting the setup described in the “Materials and methods” section (a SEM image and the measured structural size of the fabricated emitters are provided in Table S1 of the Supporting information). This indicates that our proposed AI-assisted method effectively fabricated the emitters. Figure 3a shows the measured I–V characteristic for nine typical emitters. We found deviations in the performance across various devices. This behavior was caused by structural variations such as the gap shown in Fig. 2b. Indeed, The Fowler–Nordheim law shows that the resulting current was determined by the gap length^74^. Table S1 shows the variation of the diameter for the fabricated CNT-nanocantilever-based emitters. It has been reported that the I–V characteristic also depends on the diameter, which is often discussed in the context of the field enhancement factor^78,79^.

To evaluate the statistical behavior of the field emission on the fabricated emitters, we introduce a performance measure for the threshold voltage *V*_th_^80^ at which a sufficient amount of the induced field-emission current *I*_th_ is observed. In this study, we focus on the case *I*_th_ = 0.10 nA. The histogram of *V*_th_ is presented in Fig. 3b. We observe a peak around *V*_th_ = 26.20, which corresponds to the median of the measured *V*_th_. As shown in Fig. 2b, the number of fabricated emitters was maximized around the designed value. The peak in Fig. 3b appears at such gap *g* and length *L*. However, several emitters were fabricated with a larger gap/length, as shown in Fig. 2a. These abnormal samples showed significantly higher threshold voltages, resulting in a histogram shift. Indeed, the average value of *V*_th_, 32.43 V, was larger than the median. Based on this observation, we also calculated the theoretical performance of the field-emission current using the Fowler–Nordheim law^42,76^; the parameters were *g* = 80 nm, *L* = 1.0 μm, and the enhancement factor was set to 1.75 to fit the peak of the histogram corresponding to *V*_th_ = 26.20 and *I*_th_ = 0.10 nA. The resulting curve is plotted in red in Fig. 3a. This theoretical curve accurately captures the trend of the measured currents of the fabricated emitters, showing that the measured currents were excited by the field emission.

### Neuromorphic computing with mass-fabricated CNT-nanocantilever-based emitters

We investigated the possibility of exploiting the mass-fabricated CNT-nanocantilever-based emitters for neuromorphic computing, e.g. a future-promising AI-based computing platform comprising a neural network. Activation functions with nonlinear behavior were implemented in this network. A unique nonlinear response of the nanoscale emitter shown in Fig. 3 motivated us to investigate the realization of this computing element using our emitters. A neural network often requires numerous functional nodes; our AI-assisted fabrication method enabled the application of the emitter directly within the neural network.

**Fig. 4:**
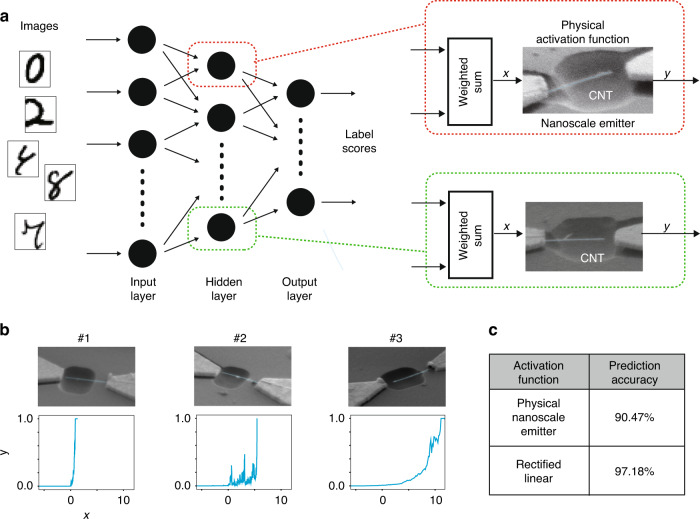
Neuromorphic computing emulated using mass-fabricated CNT-nanocantilever-based emitters. **a** Framework of the introduced Neuromorphic computing. These emitters physically implement the activation function, which is a crucial component of a neural network. The nonlinear response of the emitters, which is shown in Fig. 3a (similar examples are also shown in **b**), enables the activation function, and the AI-assisted fabrication method allowed us to introduce this concept for many emitters. Note that the handwritten images were obtained from ref. ^81^. **b** Examples of the nonlinear response of the emitters. Due to structural variations, deviations in the responses were observed. In our numerical simulation with these responses, the range of the input *x* and output *y* were normalized to fit the range of values across the layers. **c** Recognition result of handwritten characters (MNIST^81^) by the neural network with physical activation function of the emitters. To evaluate these performances, we physically emulated the neural network with the nonlinear response by using experimental data (see “Materials and methods”). The prediction accuracy of our proposed method for a given input image was comparable to the result of a popular activation function, the Rectified linear unit function^73^

The neural network employed in this study contained a three-layered structure, as shown in Fig. 4a. An image recognition problem was considered; in a typical recognition problem involving handwritten characters such as those in MNIST^81^, the network guesses the character that has been provided as an input. The notable point is that we built nodes containing an activation function in the hidden layer by using the mass-fabricated emitters (Fig. 4b). We thus evaluated the effectiveness of this neural network in recognizing the handwritten characters^81^. For this evaluation, we emulated the neural network by using the experimental data on a computer; we evaluated the recognition performance and compared it with that obtained by the neural network using the traditional rectified linear function^73^. The details of this emulation are provided in the “Materials and methods” section. The number of nodes in the input layer was the same as the size of the handwritten input image, that is, 784 (=28 × 28 pixels). The hidden layer contained 300 nodes, and we obtained the score for every ten digits (0 to 9) from the output layer with ten nodes. The digit with the highest score was determined to be the input character.

The obtained prediction accuracy is shown in Fig. 4c. The rectified linear activation function achieved a prediction accuracy of 97.18%. For this reference performance, the proposed function with the emitter achieved a prediction accuracy of 90.47%. Thus, the prediction accuracy of our method was comparable to the result of the popular rectified linear activation function. This establishes the utility of our emitter as a device for realizing an activation function.

## Conclusion

We reported an AI-assisted fabrication method for the development of devices with a CNT-based nanocantilever. To achieve efficient fabrication, we utilized randomly positioned single CNTs on the substrate and trained a deep neural network to recognize the single CNTs and measure their positions accurately without manual operation. Based on the original dataset, the trained neural network also provided information on the edge on which the CNT should be clamped to form its nanocantilever. We successfully fabricated more than 34 CNT-nanocantilever-based field emitters in one fabrication process. This platform achieved a small recognition and measurement error: the trained network detected the correct position for 90% of the recognized CNTs within an error of 200 nm. With the proposed framework, the recognition and measurement processes were automatically completed in 2 s, compared to a manual process that required 12 h. Thus, our AI-assisted method significantly reduces costly manual procedures, establishes accurate fabrication of CNT-based nanocantilevers, and shortens the time required to locate the CNTs and design the electrodes over them. Indeed, we demonstrated that the average length and gap of the fabricated nanocantilevers agreed well with the designed value, such that a field-emission current was obtained with a low applied voltage. Further improvements on the field-emission current might be achieved by using single CNTs with a prespecified diameter value. To further control the diameter of the cantilever tip, we will consider incorporating a method in the preparation step before AI-assisted fabrication. Using randomly-positioned CNTs with a specific diameter size, the AI-assisted fabrication realizes the CNT-nanocantilever-based field emitters with the designed cantilever diameter. If a high-definition image is available to see the size of the diameter, the developed AI might also pick up only the CNTs with a designed diameter.

We also showed the capability of neuromorphic computing using mass-fabricated CNT-nanocantilever-based field emitters. The physical realization of the activation function, which is one of the key functions in neural networks, was emulated using the individual CNT-based field emitter. The neural network with the CNT-nanocantilever-based emitters successfully recognized handwritten images. Such nonlinear characteristics found in the CNT-nanocantilever-based emitters are observed in many devices, for example, semiconductor-based devices such as diodes and transistors. Using the CNT-nanocantilever-based field emitter offers additional merit in terms of sensing; a resonantly driven nanomechanical vibration of the CNT tip can perform ultra-sensitive detection of various physical quantities (for example, force, mass, and electric/magnetic spin). Such platforms have led to the development of a wide range of applications, including chemical/biological/inertial sensors, nanomechanical computing, and quantum information science. Our proposed method can also be used to fabricate massive double-clamped systems, including nanobeams. We, therefore, suggest that our method can accelerate research and development on CNT-based field emitters and nanomechanical systems.

## Materials and methods

### Fabrication of CNT-based nanocantilever

We prepared a silicon substrate of size 400 mm^2^ (20 mm × 20 mm). This substrate was coated by a 3 μm-thick silicon dioxide layer, which was fabricated using plasma-enhanced chemical vapor deposition. With this substrate, we first created the Ti/Au markers using EB lithography and deposition.

We used straight CNTs produced by the arc discharge method. These CNTs were dispersed in IPA and tangled. For preparing the randomly positioned CNTs on the substrate with the markers, these CNTs were first dispersed through ultrasonic dispersion. The IPA with the CNTs was then dropped on the substrate and quickly evaporated on a heating plate. As a result, single CNTs were randomly positioned on the substrate, as shown in Fig. 1a. With the proposed AI-assisted method, we automatically obtained the pattern of the electrodes in a CAD file. The 110-nm-thick Ti/Au electrodes were fabricated using the CAD file through EB lithography and deposition. To form a hole around the nanocantilever, we patterned a rectangular etching area centered at (*X*_Hole_, *Y*_Hole_) using EB lithography. The details of the shape and size are shown in Fig. S2b in the Supporting information. This central position was calculated using (3) based on the information obtained from Faster R-CNN. The silicon dioxide under the CNT was then etched with buffered hydrofluoric acid. We finally prepared the nanocantilever by drying the substrate with a supercritical dryer.

### Dataset and training of Faster R-CNN

The CNN contained in the Faster R-CNN model was previously learned by VGG16^82,83^. For tuning the CNT recognition capability, we trained the entire network with the originally created dataset, which contains the coordinates of the two corners (top-left and bottom-right), (*x*_*⌜*_, *y*_*⌜*_) and (*x*_*⌟*_, *y*_*⌟*_), of the bounding box surrounding the CNT or marker found in each SEM image. The details are shown in Fig. S1 in the Supporting information. As described in Fig. 1(b), the unit of the coordinates is a pixel, and the origin is located at the top-left center of each SEM image. The entry with the label “M” provides the position of the marker. In the other cases, the label ∈{"*⌜*”, “*⌝*”, “*⌞*”, “*⌟*”} shows the preferred direction for the anode in the fabrication of the electrodes. Here, “*⌜*” is top-left, “*⌝*” is top-right, “*⌞*” is bottom-left, and “*⌟*” is bottom-right of the bounding box; for example, the label “*⌜*” indicates that the fabrication around the top-left point is preferable. We manually inspected 1404 SEM images, found 2080 CNTs in the images, and then obtained information (coordinates and labels) to create the dataset. Again, this type of dataset enabled the automatic selection of the preferred edge for placing the cathode and anode.

During training, each SEM image in the dataset was input to the CNN. Simultaneously, the rest of the data in the entry, (*x*_*⌜*_, *y*_*⌜*_), (*x*_*⌟*_, *y*_*⌟*_), and the label, were also input onto the output side of the network. With these data, the Faster R-CNN model studied 500 epochs with the optimizer of momentum SGD^84^. The learning rate was changed from 0.001 to 0.0001 after 375 epochs for efficient learning. We used 1311 SEM images for the learning, and the remaining 93 SEM images were used for testing. A weight decay of 0.0005 was applied to regularize the model. To run the Faster R-CNN for learning/testing, we used the free source code provided in ref. ^85^. The training process was executed on a GPU (NVIDIA Tesla K80).

### Determination of positions of nanocantilever and hole

To determine the positions of the nanocantilever and hole, we first calculated the relative distances in meters between the two edges of the recognized CNT and the marker in each SEM image. These distances, *x*_a_, *y*_a_, *x*_c_, and *y*_c_ which are shown in Fig. S2a in the Supporting information, were calculated using the network output (*x*_*⌜*_, *y*_*⌜*_) and (*x*_*⌟*_, *y*_*⌟*_) and the corresponding labels. In this calculation, we considered a fixed scale to convert the pixels to meters (7.143 nm per pixel in this study).

The fabrication process was conducted according to a common coordinate on the substrate, whose origin was positioned at the lower-left corner. We first converted the measured position into those in terms of the common coordinate. We know that the position of the closest marker in the common coordinate is (*X*_M_, *Y*_M_); hence, the two measured positions were converted into (*X*_a_, *Y*_a_) = (*X*_M_ + *x*_a_, *Y*_M_ + *y*_a_) and (*X*_c_, *Y*_c_) = (*X*_M_ + *x*_c_, *Y*_M_ + *y*_c_), as shown in Fig. S2 in the Supporting information. These positions were then used to design the two electrodes (anode and cathode). By using the preset parameters, namely, the gap *g* between the tip of the CNT and anode surface, length of the emitter *L*, and distance from the edge to the center of electrode ${l}_{{{{\rm{electr}}}}}$, the central positions of the two electrodes are calculated as

1 $$({X}_{{{{\rm{Anode}}}}},{Y}_{{{{\rm{Anode}}}}})=\left({X}_{{{{\rm{a}}}}}+(g+{l}_{{{{\rm{electr}}}}})\cos \theta ,{Y}_{{{{\rm{a}}}}}+(g+{l}_{{{{\rm{electr}}}}})\sin \theta \right),$$

2 $$({X}_{{{{\rm{Cathode}}}}},{Y}_{{{{\rm{Cathode}}}}})=\left({X}_{{{{\rm{a}}}}}-(L+{l}_{{{{\rm{electr}}}}})\cos \theta ,{Y}_{{{{\rm{a}}}}}-(L+{l}_{{{{\rm{electr}}}}})\sin \theta \right),$$

where $\sin \theta =({X}_{{{{\rm{a}}}}}-{X}_{{{{\rm{c}}}}})/\sqrt{{({X}_{{{{\rm{a}}}}}-{X}_{{{{\rm{c}}}}})}^{2}+{({Y}_{{{{\rm{a}}}}}-{Y}_{{{{\rm{c}}}}})}^{2}}$ and $\cos \theta =({Y}_{{{{\rm{a}}}}}-{Y}_{{{{\rm{c}}}}})/\sqrt{{({X}_{{{{\rm{a}}}}}-{X}_{{{{\rm{c}}}}})}^{2}+{({Y}_{{{{\rm{a}}}}}-{Y}_{{{{\rm{c}}}}})}^{2}}$. The shape of the electrode can be obtained for a given angle *θ* and center position, which can then be drawn in the CAD file.

To create a hole around the nanocantilever, we patterned the rectangular etching area centered at (*X*_Hole_, *Y*_Hole_) using EB lithography. This etching point was located at the middle point between the centers of the two electrodes.

3 $$({X}_{{{{\rm{Hole}}}}},{Y}_{{{{\rm{Hole}}}}})=\left(\frac{{X}_{{{{\rm{Anode}}}}}+{X}_{{{{\rm{Cathode}}}}}}{2},\frac{{Y}_{{{{\rm{Anode}}}}}+{Y}_{{{{\rm{Cathode}}}}}}{2}\right)$$

### Setup for measurement of I–V characteristics of CNT-nanocantilever-based emitter

The I–V characteristics of the CNT-nanocantilever-based field emitters was measured in a high-vacuum chamber with a vacuum pressure of 1.0 × 10^−5^ Pa. The cathode and anode of the emitter were electrically contacted with metal probes. We adjusted the position of the tip of the probes by using a four-axis stage controller (Sigmakoki, VSGSP60(XY), VSGSP60(Z), and SHOT-304GS) to secure the contact. Those probes were connected to an electrometer (Keithley, model 6430) to apply the bias voltage and measure the resulting field-emission current.

### Emulation and training of neural network with physical activation function

We emulated the neural network using the activation function of the emitter based on the experimental data. We built the neural network model on a GPU with PyTorch^86^. The activation function used here was originally defined according to the I–V data measured on the fabricated nanoscale emitters. Some examples of the function are shown in Fig. 4b; to derive them, we first applied a logarithm for linear interpolation and exponentiated the current on the measured data. We then shifted the obtained I–V data such that the voltage of 0 V corresponded to the point where the current began increasing exponentially. To fit the range of the current and voltage with the input/output of the upper/lower layers, we normalized the range of the input current (*x*) and output voltage (*y*). In this manner, we prepared 35 functions from the set of measured data. These functions were randomly selected and assigned to each node in the hidden layer to evaluate the prediction accuracy in the image recognition problem. Note that we can perform operations such as shifting and scaling with practical devices; for example, scaling can be executed with an attenuator and/or amplifier.

We used the MNIST dataset in the training process; with these data, the neural network model studied 500 epochs with the stochastic optimizer Adam^86^. We changed the learning rate from 0.001 to 0.0001 after 450 epochs for efficient learning. We used 60,000 images for the learning and 10,000 images for the testing.

## Supplementary information

Supporting information

## Data Availability

The data that support the findings of this study are available from the corresponding author on reasonable request.

## References

[1] Binnig G, Quate CF, Gerber C (1986). Atomic force microscope. Phys. Rev. Lett..

[2] Michels T, Rangelow IW (2014). Review of scanning probe micromachining and its applications within nanoscience. Microelectron. Eng..

[3] Leung C (2012). Atomic force microscopy with nanoscale cantilevers resolves different structural conformations of the DNA double helix. Nano Lett..

[4] Cha W (2021). Hollow atomic force microscopy cantilevers with nanoscale wall thicknesses. Small.

[5] Dykman, M. I. *Fluctuating Nonlinear Oscillators: From Nanomechanics to Quantum Superconducting Circuits* (Oxford, 2012).

[6] Schmid, S., Villanueva, L. G. & Roukes, M. L. *Fundamentals of Nanomechanical Resonators* (Springer, 2016).

[7] Maillet O (2018). Measuring frequency fluctuations in nonlinear nanomechanical resonators. ACS Nano.

[8] Bachtold A, Moser J, Dykman MI (2022). Mesoscopic physics of nanomechanical systems. Rev. Mod. Phys..

[9] Yasuda M, Takei K, Arie T, Akita S (2016). Oscillation control of carbon nanotube mechanical resonator by electrostatic interaction induced retardation. Sci. Rep..

[10] Papariello L, Zilberberg O, Eichler A, Chitra R (2016). Ultrasensitive hysteretic force sensing with parametric nonlinear oscillators. Phys. Rev. E.

[11] Chaste J (2012). A nanomechanical mass sensor with yoctogram resolution. Nat. Nanotechnol..

[12] Łabȩdź B, Wańczyk A, Rajfur Z (2017). Precise mass determination of single cell with cantilever-based microbiosensor system. PLoS ONE.

[13] Tao Y, Eichler A, Holzherr T, Degen CL (2016). Ultrasensitive mechanical detection of magnetic moment using a commercial disk drive write head. Nat. Commun..

[14] Arash B, Jiang J-W, Rabczuk T (2015). A review on nanomechanical resonators and their applications in sensors and molecular transportation. Appl. Phys. Rev..

[15] Eom K, Park HS, Yoon DS, Kwon T (2011). Nanomechanical resonators and their applications in biological/chemical detection: nanomechanics principles. Phys. Rep..

[16] Tamayo J, Kosaka PM, Ruz JJ, San Paulo A, Calleja M (2013). Biosensors based on nanomechanical systems. Chem. Soc. Rev..

[17] Ruz JJ, Tamayo J, Pini V, Kosaka PM, Calleja M (2014). Physics of nanomechanical spectrometry of viruses. Sci. Rep..

[18] Wenzler J-S, Dunn T, Toffoli T, Mohanty P (2014). A nanomechanical fredkin gate. Nano Lett..

[19] Coulombe JC, York MCA, Sylvestre J (2017). Computing with networks of nonlinear mechanical oscillators. PLoS ONE.

[20] Chappanda KN (2017). A single nano cantilever as a reprogrammable universal logic gate. J. Micromech. Microeng..

[21] Dion G, Mejaouri S, Sylvestre J (2018). Reservoir computing with a single delay-coupled non-linear mechanical oscillator. J. Appl. Phys..

[22] Sudhir V (2017). Quantum correlations of light from a room-temperature mechanical oscillator. Phys. Rev. X.

[23] Degen CL, Reinhard F, Cappellaro P (2017). Quantum sensing. Rev. Mod. Phys..

[24] Pistolesi F, Cleland AN, Bachtold A (2021). Proposal for a nanomechanical qubit. Phys. Rev. X.

[25] Rangelow IW (1994). Sharp silicon tips for AFM and field emission. Microelectronic Eng..

[26] Wisitsora-at A (2003). High current diamond field emission diode. J. Vac. Sci. Technol. B Microelectron. Nanometer Struct. Process. Meas. Phenom..

[27] Jensen KL (2003). Electron emission theory and its application: Fowler-Nordheim equation and beyond. J. Vac. Sci. Technol. B Microelectron. Nanometer Struct. Process. Meas. Phenom..

[28] Zhang H (2016). An ultrabright and monochromatic electron point source made of a lab6 nanowire. Nat. Nanotechnol..

[29] Liu M, Fu W, Yang Y, Li T, Wang Y (2018). Excellent field emission properties of vo2(a) nanogap emitters in air. Appl. Phys. Lett..

[30] Chen Y (2018). Investigation of the temperature dependent field emission from individual ZnO nanowires for evidence of field-induced hot electrons emission. J. Phys. Condens. Matter.

[31] Jiang R (2022). Design of a ka-band traveling wave tube using low turn-on field emission electron source made by carbon nanotubes. IEEE Trans. Plasma Sci..

[32] Lee YZ (2011). Carbon nanotube based x-ray sources: applications in pre-clinical and medical imaging. Nuclear Instrum. Methods Phys. Res. A.

[33] Chen S, Yang W (2017). Flexible low-dimensional semiconductor field emission cathodes: fabrication, properties and applications. J. Mater. Chem. C.

[34] Zhang H (2016). An ultrabright and monochromatic electron point source made of a lab6 nanowire. Nat. Nanotechnol..

[35] Moser J (2013). Ultrasensitive force detection with a nanotube mechanical resonator. Nat. Nanotechnol..

[36] Moser J, Eichler A, Güttinger J, Dykman MI, Bachtold A (2014). Nanotube mechanical resonators with quality factors of up to 5 million. Nat. Nanotechnol..

[37] Descombin A (2019). Giant, voltage tuned, quality factors of single wall carbon nanotubes and graphene at room temperature. Nano Lett..

[38] Jung JE (2002). Fabrication of triode-type field emission displays with high-density carbon-nanotube emitter arrays. Phys. B Condens. Matter.

[39] Dwivedi N (2021). The rise of carbon materials for field emission. J. Mater. Chem. C.

[40] Atakan B, Akan O (2010). Carbon nanotube-based nanoscale ad hoc networks. IEEE Commun. Mag..

[41] Tadokoro Y, Tanaka H, Dykman MI (2018). Driven nonlinear nanomechanical resonators as digital signal detectors. Sci. Rep..

[42] Funayama K (2021). Carbon nanotube-based nanomechanical receiver for digital data transfer. ACS Appl. Nano Mater..

[43] Akyildiz IF, Kak A, Nie S (2020). 6g and beyond: the future of wireless communications systems. IEEE Access.

[44] Engel M (2018). Graphene-enabled and directed nanomaterial placement from solution for large-scale device integration. Nat. Commun..

[45] Corletto A, Shapter JG (2021). Nanoscale patterning of carbon nanotubes: techniques, applications, and future. Adv. Sci..

[46] Rao R (2018). Carbon nanotubes and related nanomaterials: critical advances and challenges for synthesis toward mainstream commercial applications. ACS Nano.

[47] Kong J, Soh HT, Cassell AM, Quate CF, Dai H (1998). Synthesis of individual single-walled carbon nanotubes on patterned silicon wafers. Nature.

[48] Purcell ST, Vincent P, Journet C, Binh VT (2002). Tuning of nanotube mechanical resonances by electric field pulling. Phys. Rev. Lett..

[49] Steele GA, Gotz G, Kouwenhoven LP (2009). Tunable few-electron double quantum dots and Klein tunnelling in ultraclean carbon nanotubes. Nat. Nanotechnol..

[50] Garcia-Sanchez D (2007). Mechanical detection of carbon nanotube resonator vibrations. Phys. Rev. Lett..

[51] Oikonomou A (2015). Scalable bottom-up assembly of suspended carbon nanotube and graphene devices by dielectrophoresis. Rapid Res. Lett..

[52] Liu L, Chen K, Xiang N, Ni Z (2019). Dielectrophoretic manipulation of nanomaterials: a review. Electrophoresis.

[53] Hofmann S, Ducati C, Kleinsorge B, Robertson J (2003). Direct growth of aligned carbon nanotube field emitter arrays onto plastic substrates. Appl. Phys. Lett..

[54] Chouhan V, Noguchi T, Kato S (2016). Field emission from optimized structure of carbon nanotube field emitter array. J. Appl. Phys..

[55] Kumar M, Okazaki T, Hiramatsu M, Ando Y (2007). The use of camphor-grown carbon nanotube array as an efficient field emitter. Carbon.

[56] Sreekanth M, Ghosh S, Srivastava P (2017). Tuning vertical alignment and field emission properties of multi-walled carbon nanotube bundles. Appl. Phys. A.

[57] Galante B, Tranquille GA, Himmerlich M, Welsch CP, Resta López J (2021). Stability and lifetime study of carbon nanotubes as cold electron field emitters for electron cooling in the CERN extra low energy antiproton ring. Phys. Rev. Accel. Beams.

[58] Bargatin I (2012). Large-scale integration of nanoelectromechanical systems for gas sensing applications. Nano Lett..

[59] Guerrera SA, Akinwande AI (2016). Nanofabrication of arrays of silicon field emitters with vertical silicon nanowire current limiters and self-aligned gates. Nanotechnology.

[60] Karaulac N, Rughoobur G, Akinwande AI (2020). Highly uniform silicon field emitter arrays fabricated using a trilevel resist process. J. Vac. Sci. Technol. B.

[61] Ren, S., He, K., Girshick, R. & Sun, J. Faster r-cnn: Towards real-time object detection with region proposal networks. In *Advances in Neural Information Processing Systems* (eds Cortes, C., Lawrence, N., Lee, D., Sugiyama, M. & Garnett, R.) (Curran Associates, Inc., 2015).

[62] Prezioso M (2015). Training and operation of an integrated neuromorphic network based on metal-oxide memristors. Nature.

[63] Marković D, Mizrahi A, Querlioz D, Grollier J (2020). Physics for neuromorphic computing. Nat. Rev. Phys..

[64] Chen F (2021). Recent progress in artificial synaptic devices: materials, processing and applications. J. Mater. Chem. C.

[65] Nichterwitz M (2021). Advances in magneto-ionic materials and perspectives for their application. APL Mater..

[66] Wang T (2022). An optical neural network using less than 1 photon per multiplication. Nat. Commun..

[67] Wright LG (2022). Deep physical neural networks trained with backpropagation. Nature.

[68] Rodrigues SP (2021). Weighing in on photonic-based machine learning for automotive mobility. Nat. Photonics.

[69] Feldmann J, Youngblood N, Wright CD, Bhaskaran H, Pernice WHP (2019). All-optical spiking neurosynaptic networks with self-learning capabilities. Nature.

[70] Marković D, Grollier J (2020). Quantum neuromorphic computing. Appl. Phys. Lett..

[71] Zahedinejad M (2020). Two-dimensional mutually synchronized spin hall nano-oscillator arrays for neuromorphic computing. Nat. Nanotechnol..

[72] Tanaka H (2018). A molecular neuromorphic network device consisting of single-walled carbon nanotubes complexed with polyoxometalate. Nat. Commun..

[73] Apicella A, Donnarumma F, Isgrò F, Prevete R (2021). A survey on modern trainable activation functions. Neural Netw..

[74] Fowler RH, Nordheim L (1928). Electron emission in intense electric fields. Proc. R. Soc. Lond. A.

[75] Tadokoro Y, Funayama K, Tanaka H (2018). Noise-enhanced field emission current from a carbon nanotube cantilever. Electron. Lett..

[76] Funayama K (2019). Dependence of enhancement factor on electrode size for field emission current from carbon nanotube on silicon wafer. Nanotechnology.

[77] gdsCAD. https://pypi.org/project/gdsCAD/ (2022).

[78] Smith RC, Carey JD, Forrest RD, Silva SRP (2005). Effect of aspect ratio and anode location on the field emission properties of a single tip based emitter. J. Vac. Sci. Technol. B Microelectron. Nanometer Struct. Process. Meas. Phenom..

[79] Parveen S, Kumar A, Husain S, Husain M (2017). Fowler Nordheim theory of carbon nanotube based field emitters. Phys. B Condens. Matter.

[80] Passacantando M (2008). Field emission from a selected multiwall carbon nanotube. Nanotechnology.

[81] LeCun, Y., Cortes, C. & Burges, C. J. The MNIST database of handwritten digits. http://yann.lecun.com/exdb/mnist/ (2022).

[82] Deng, J. et al. Imagenet: a large-scale hierarchical image database. In *2009 IEEE Conference on Computer Vision and Pattern Recognition* 248–255 (IEEE, 2009).

[83] Simonyan, K. & Zisserman, A. Very deep convolutional networks for large-scale image recognition. In *2015 International Conference on Learning Representations* (2015).

[84] Sutskever, I., Martens, J., Dahl, G. & Hinton, G. On the importance of initialization and momentum in deep learning. In *Proceedings of the 30th International Conference on Machine Learning* 1139–1147 (PMLR, 2013).

[85] chenyuntc/simple-faster-rcnn-pytorch. https://github.com/chenyuntc/simple-faster-rcnn-pytorch (2022).

[86] Pytorch. https://pytorch.org/ (2022).

