# Supplementary material for: Artificial-intelligence-assisted mass fabrication of nanocantilevers from randomly positioned single carbon nanotubes
Source: Microsyst Nanoeng. 2023 Mar 22;9:32. doi: 10.1038/s41378-023-00507-1 (PMC10033894; doi:10.1038/s41378-023-00507-1)
Supplement: Supplementary file 1 — Supporting information [file 41378_2023_507_MOESM1_ESM.docx]

Supporting information for “Artificial-intelligence-assisted mass

fabrication of nanocantilevers from randomly positioned single carbon nanotubes”

Yukihiro Tadokoro,*^∗^*^,^*^†^*^,^*^¶^* Keita Funayama,*^∗^*^,^*^†^* Keisuke Kawano,*^†^* Atsushi Miura,*^†^* Jun Hirotani,*^‡^* Yutaka Ohno,*^‡^* and Hiroya Tanaka*^†^*

*†Toyota Central R&D Labs., Inc. Nagakute, Aichi, 480-1192, Japan*

*‡Nagoya University, Nagoya, Aichi, Japan*

*¶Toyota Research Institute of North America, Ann Arbor, MI, 48105, USA*

E-mail: y.tadokoro@ieee.org; [funayama@mosk.tytlabs.co.jp](mailto:funayama@mosk.tytlabs.co.jp)

**Supporting figures and table**


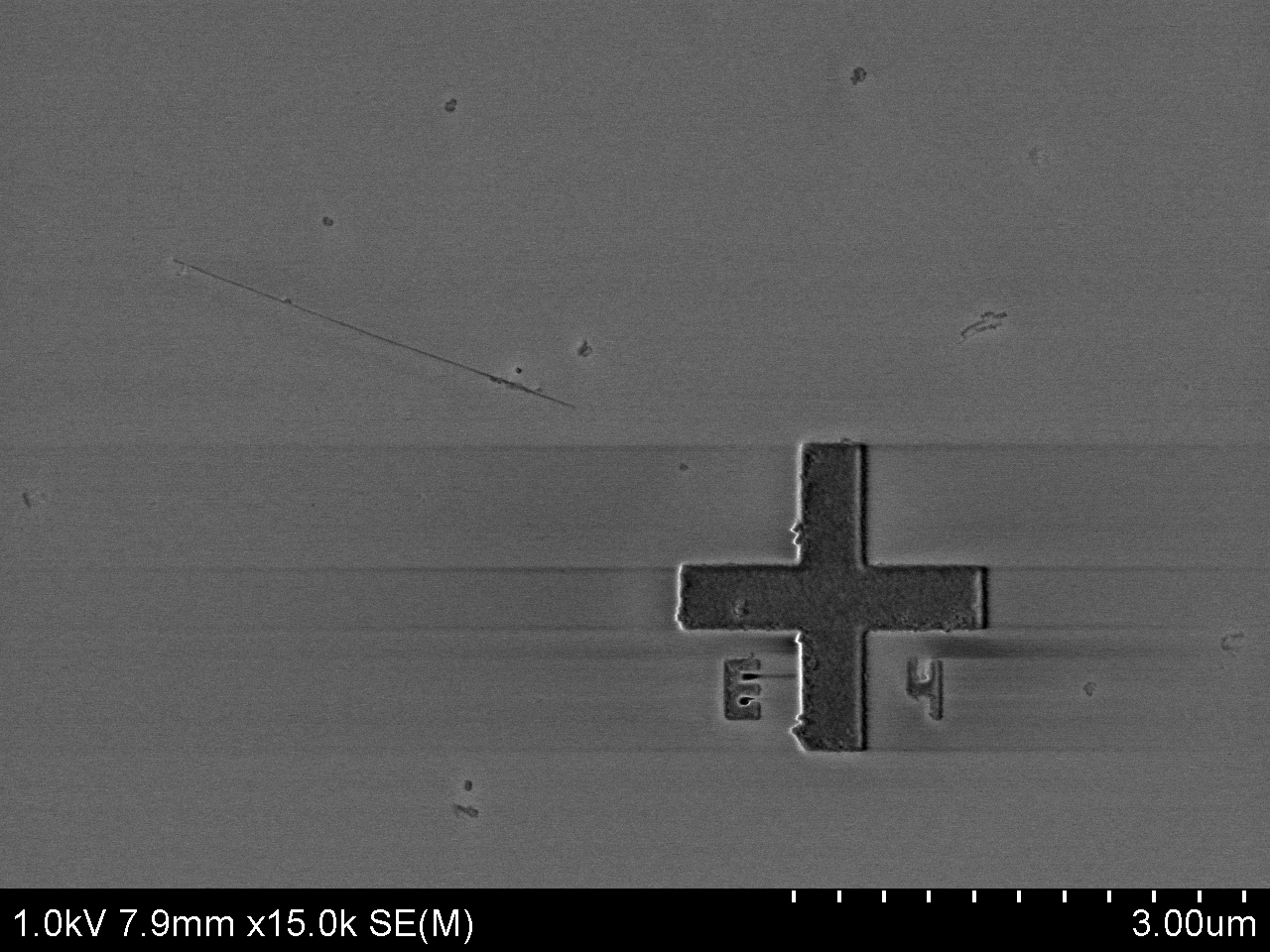
SEM image

Dataset


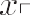

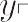

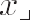


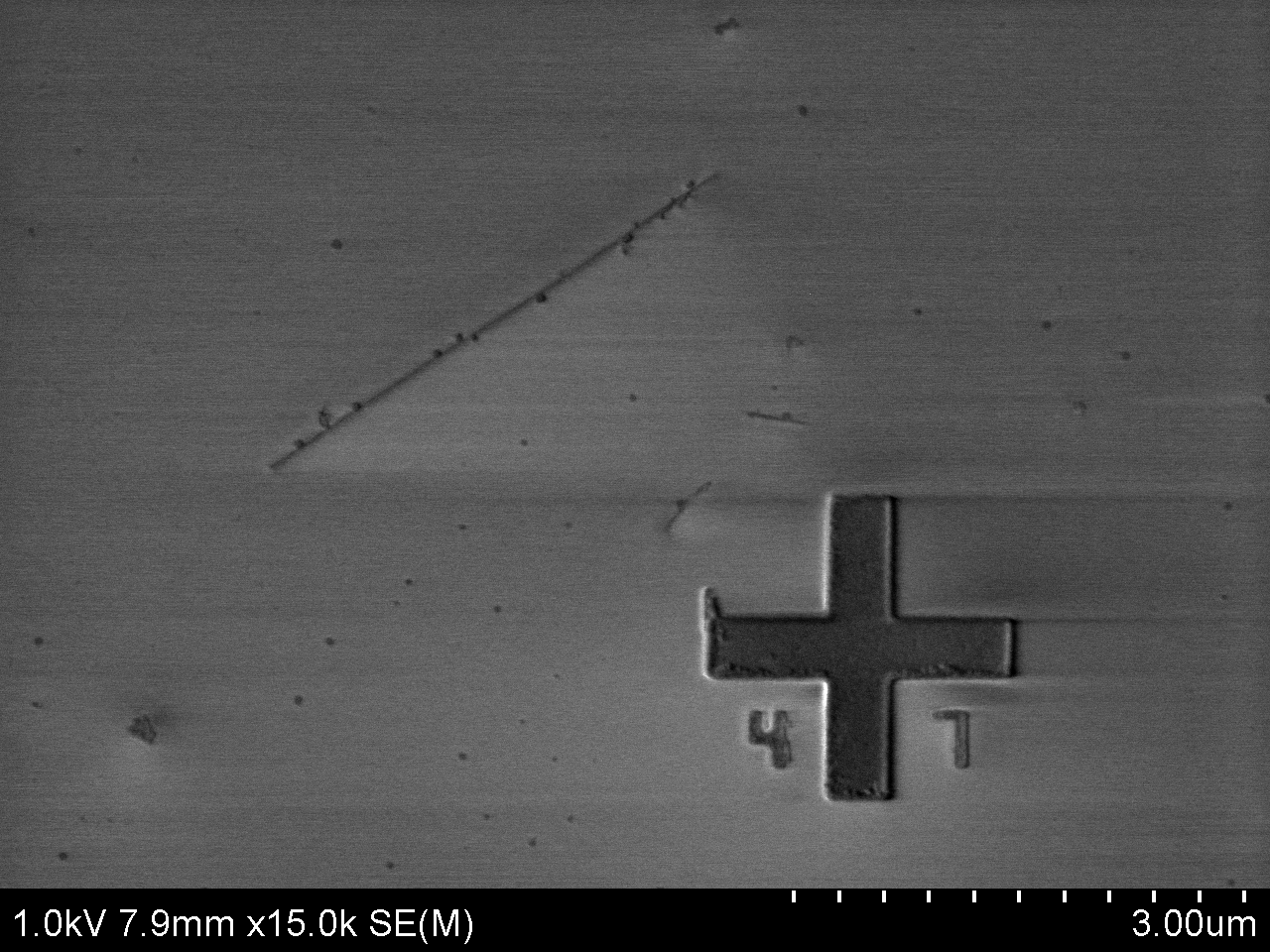

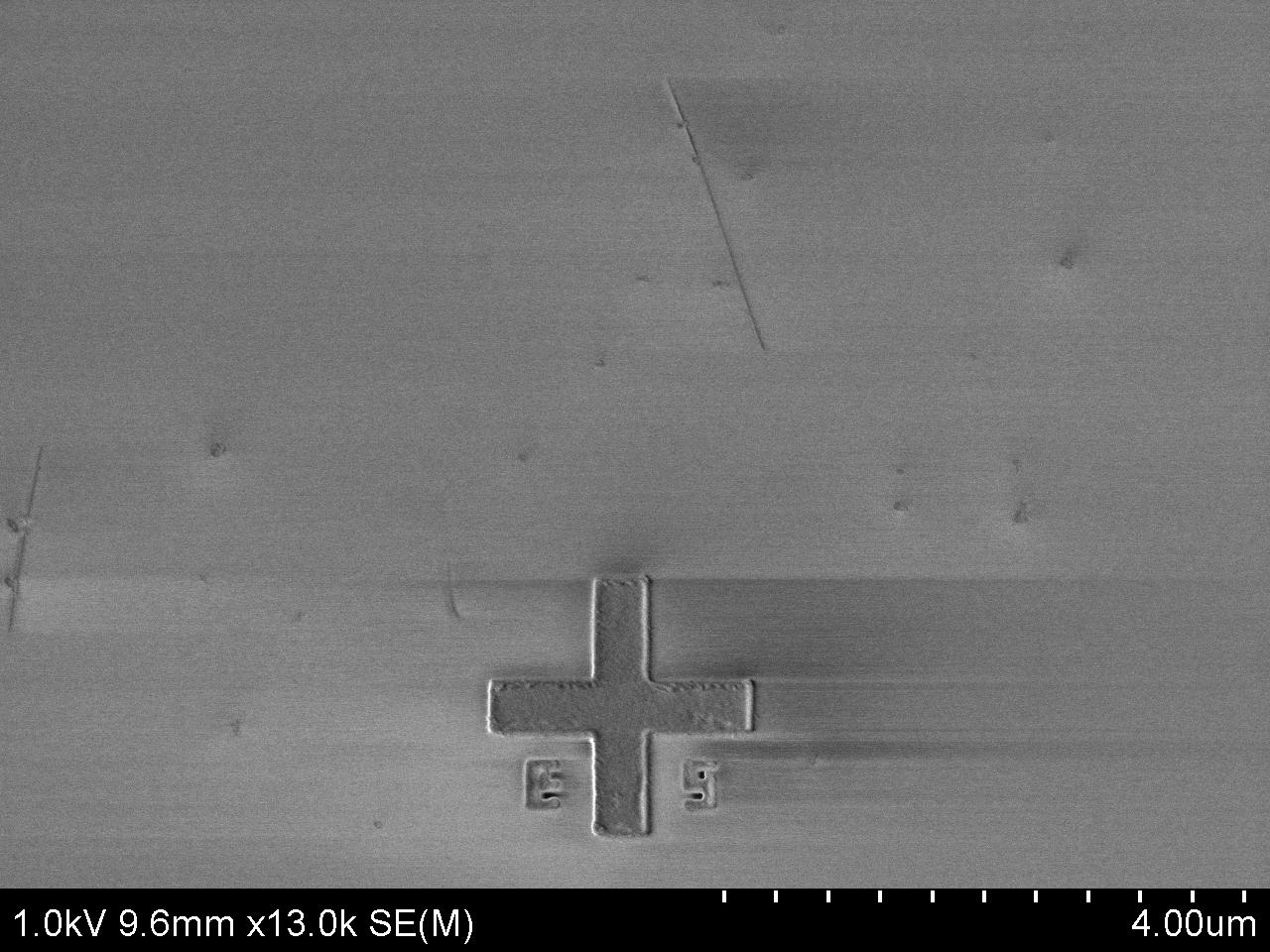

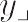
 Label

| #1 | 684 | 448 | 990 | 756 | M |
| --- | --- | --- | --- | --- | --- |
|  | 175 | 261 | 559 | 407 |  |
| #2 | 709 | 499 | 1020 | 807 | M |
|  | 273 | 490 | 719 | 177 |  |
|  |  |  |  |  |  |
| #1404 | 491 | 582 | 758 | 845 | M |
|  | 672 | 80 | 770 | 352 |  |


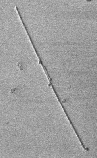

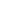

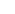


Anode


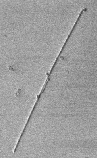

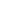

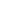

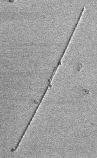

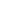

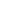

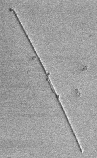

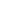

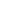

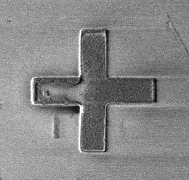

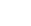

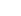

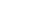

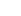

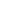

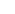


Label = ‘ ’ Label = ‘ ’ Label = ‘ ’ Label = ‘ ’ Label = ‘M’

Figure S1: Structure of dataset used for training and five types of the labels.

**(a)**

Anode , Anode

a, a

a

# Anode

electr


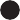

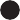

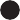

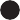

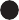

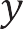

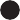

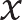

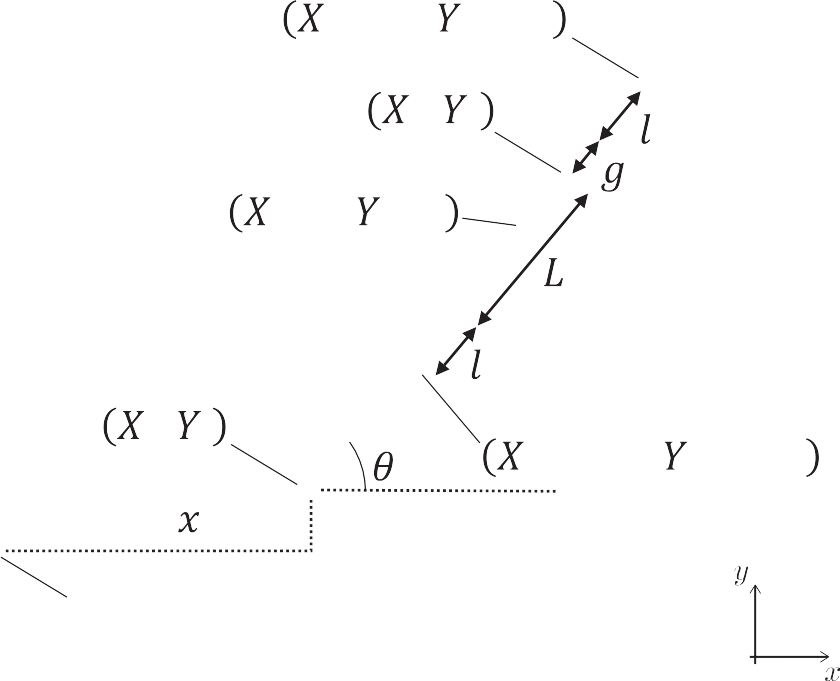

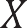

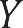


Hole , Hole

# Cathode


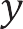
a

electr

c , c

,

Cathode Cathode

# Marker

c c

M , M

**(b)**

Anode , Anode


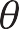

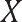

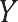

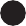

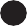

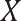

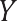

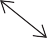

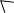

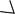

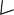

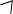

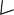

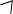

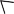

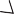

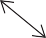

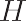

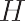

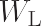

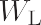

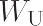


= 1000 nm

# Anode

CNT

= 300 nm

= 1000 nm

Cathode , Cathode

= 1000 nm

= 1000 nm

# Cathode

Figure S2: (a) Calculation of emitter and hole positions based on recognized CNTs. The parameters *L* = 1.0 *µ*m, *g* = 80 nm, and *l*_electr_ = 500 nm were used in our fabrication pro- cess. (b) Size of the designed trapezoidal electrodes. The two electrodes are centered at (*X*_Anode_*, Y*_Anode_) or (*X*_Cathode_*, Y*_Cathode_). The two widths and height are *W*_U_ = 300 nm, *W*_L_ = 1000 nm, and *H* = 1000 nm.

Table S1: SEM image and measured structural size of fabricated 34 CNT-nanocantilevers.

| No. | SEM image | Length (*µ*m) | Gap (*µ*m) | Diameter (nm) |
| --- | --- | --- | --- | --- |
| 1 | 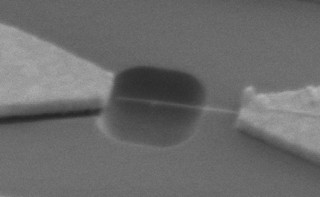 | 0.95238 | 0.12143 | 18.2 |
| 2 | 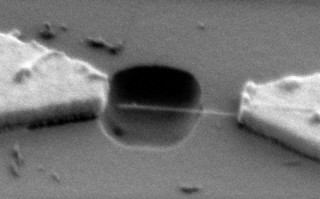 | 0.73651 | 0.22857 | 22.7 |
| 3 | 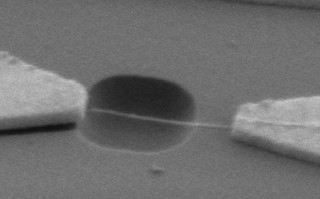 | 1.01587 | 0.15000 | 15.9 |
| 4 | 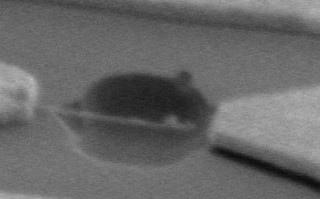 | 0.76190 | 0.20714 | 20.5 |
| 5 | 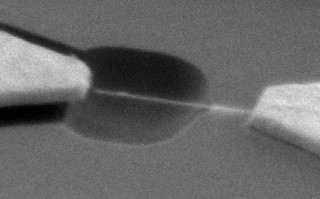 | 1.06349 | 0.01190 | 15.9 |
| Continue to the next page | | | | |

| No. | SEM image | Length (*µ*m) | Gap (*µ*m) | Diameter (nm) |
| --- | --- | --- | --- | --- |
| 6 | 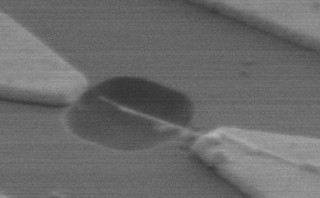 | 0.69841 | 0.25476 | 20.5 |
| 7 | 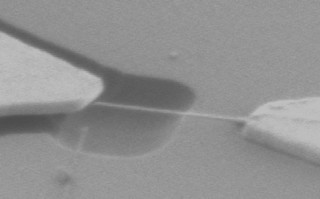 | 1.09524 | 0.10714 | 27.3 |
| 8 | 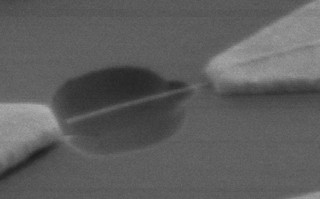 | 0.90476 | 0.10714 | 25.0 |
| 9 | 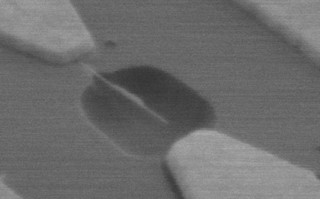 | 0.66667 | 0.28571 | 22.7 |
| 10 | 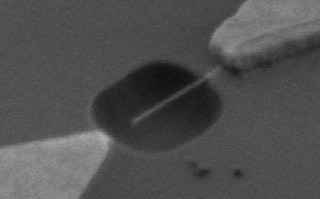 | 0.78730 | 0.22143 | 20.5 |
| Continue to the next page | | | | |

| No. | SEM image | Length (*µ*m) | Gap (*µ*m) | Diameter (nm) |
| --- | --- | --- | --- | --- |
| 11 | 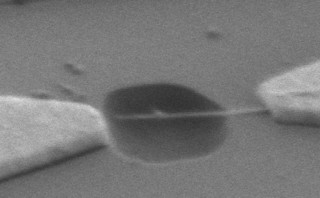 | 0.92063 | 0.11905 | 15.9 |
| 12 | 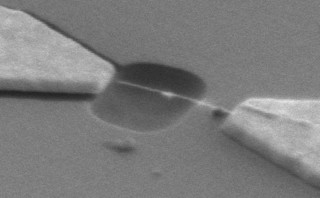 | 0.96825 | 0.07381 | 13.6 |
| 13 | 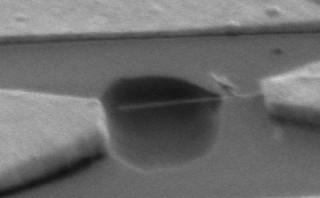 | 0.90476 | 0.19286 | 13.6 |
| 14 | 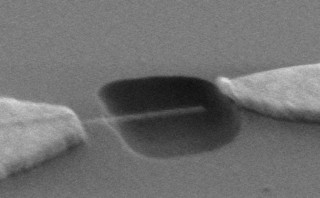 | 0.86349 | 0.23810 | 20.5 |
| 15 | 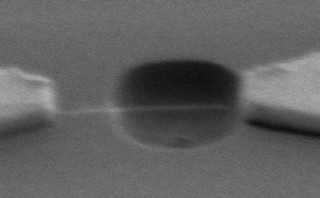 | 0.98095 | 0.07857 | 11.4 |
| Continue to the next page | | | | |

| No. | SEM image | Length (*µ*m) | Gap (*µ*m) | Diameter (nm) |
| --- | --- | --- | --- | --- |
| 16 | 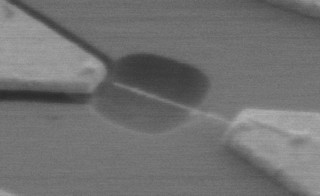 | 0.88254 | 0.11905 | 18.2 |
| 17 | 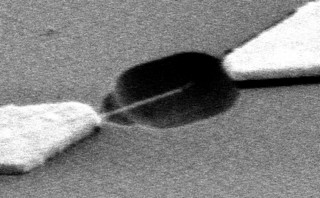 | 0.66055 | 0.40367 | 13.6 |
| 18 | 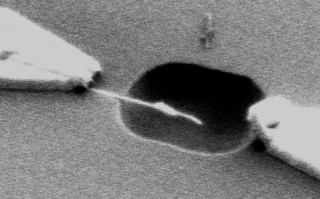 | 0.73394 | 0.35596 | 25.0 |
| 19 | 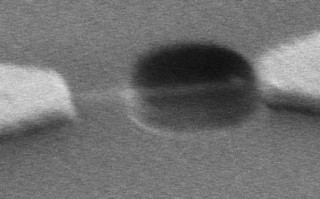 | 0.93016 | 0.10476 | 18.2 |
| 20 | 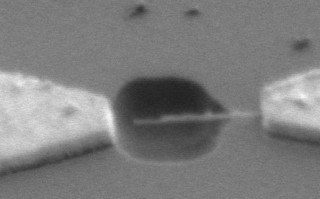 | 0.77143 | 0.21429 | 20.5 |
| Continue to the next page | | | | |

| No. | SEM image | Length (*µ*m) | Gap (*µ*m) | Diameter (nm) |
| --- | --- | --- | --- | --- |
| 21 | 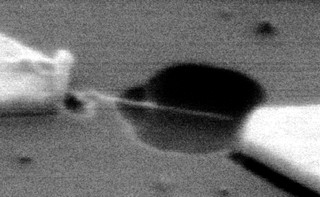 | 0.93333 | 0.08333 | 11.4 |
| 22 | 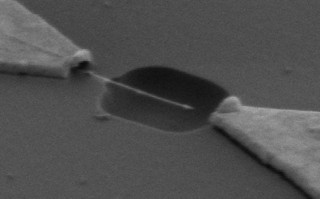 | 0.85714 | 0.15000 | 13.6 |
| 23 | 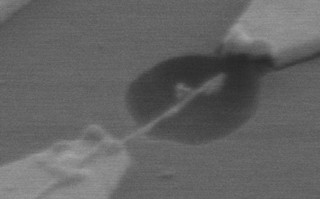 | 1.00000 | 0.14286 | 25.0 |
| 24 | 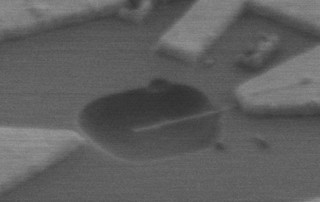 | 0.53968 | 0.38333 | 18.2 |
| 25 | 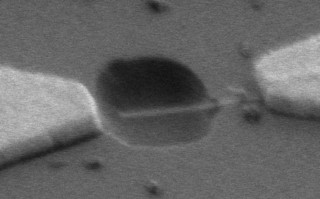 | 0.84762 | 0.12857 | 20.5 |
| Continue to the next page | | | | |

| No. | SEM image | Length (*µ*m) | Gap (*µ*m) | Diameter (nm) |
| --- | --- | --- | --- | --- |
| 26 | 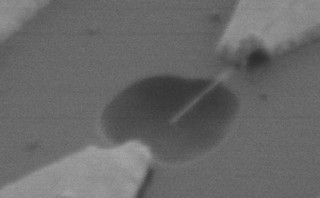 | 0.77921 | 0.32619 | 15.9 |
| 27 | 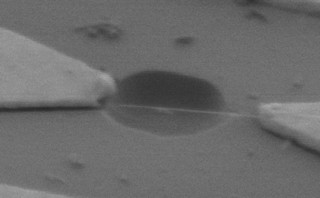 | 1.01587 | 0.02381 | 11.4 |
| 28 | 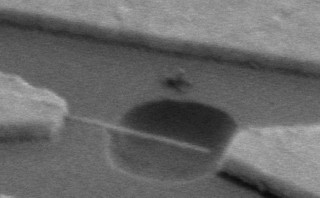 | 0.92063 | 0.08333 | 25.0 |
| 29 | 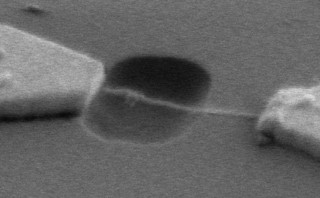 | 1.01587 | 0.04762 | 27.3 |
| 30 | 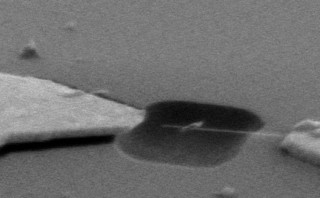 | 0.88794 | 0.20524 | 9.1 |
| Continue to the next page | | | | |

| No. | SEM image | Length (*µ*m) | Gap (*µ*m) | Diameter (nm) |
| --- | --- | --- | --- | --- |
| 31 | 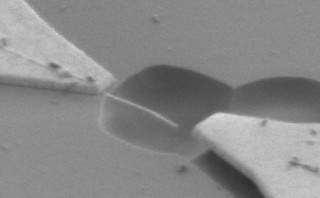 | 0.89524 | 0.19048 | 27.3 |
| 32 | 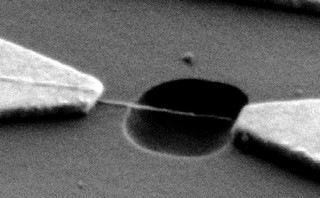 | 1.04762 | 0.03810 | 15.9 |
| 33 | 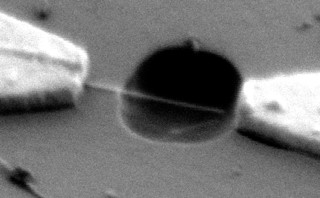 | 0.98413 | 0.09524 | 11.4 |
| 34 | 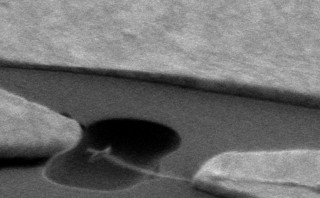 | 0.85714 | 0.20714 | 22.7 |
| End of table | | | | |
